# Health-Related Quality of Life and Physical Function in Individuals with Parkinson’s Disease after a Multidisciplinary Rehabilitation Regimen—A Prospective Cohort Feasibility Study

**DOI:** 10.3390/ijerph17207668

**Published:** 2020-10-21

**Authors:** Christina Nielsen, Volkert Siersma, Emma Ghaziani, Nina Beyer, S. Peter Magnusson, Christian Couppé

**Affiliations:** 1 Department of Physical and Occupational Therapy, Bispebjerg Hospital, 2400 Copenhagen, Denmark; emma.ghaziani@regionh.dk (E.G.); nb01@bbh.regionh.dk (N.B.); p.magnusson@sund.ku.dk (S.P.M.); christian.couppe@regionh.dk (C.C.); 2 Institute of Sports Medicine Copenhagen, Department of Orthopaedic Surgery M, Bispebjerg Hospital and Center for Healthy Aging, Faculty of Health and Medical Sciences, University of Copenhagen, 2400 Copenhagen, Denmark; 3 Research Unit and Department of General Practice, Institute of Public Health, University of Copenhagen, 1014 Copenhagen, Denmark; siersma@sund.ku.dk

**Keywords:** multidisciplinary rehabilitation, health-related quality of life, physical function, Parkinson’s disease

## Abstract

Parkinson’s disease (PD) is a neurodegenerative disease and a multidisciplinary approach to rehabilitation has been suggested as the best clinical practice. However, very few studies have investigated the long-term effects of a multidisciplinary rehabilitation approach, particularly regarding whether this can slow the progression of PD. The purpose was to investigate the short- and long-term effect of a 2-week multidisciplinary rehabilitation regimen on the PD-related decline in health-related quality of life (HRQOL), mobility, and muscle function. Individuals with PD (IPD) participated in a 2-week inpatient multidisciplinary rehabilitation regimen that focused on improving HRQOL, mobility, and muscle function. Data from the primary outcome: HRQOL (Parkinson’s Disease Questionnaire 39, PDQ-39), secondary outcomes: handgrip strength, Timed-up and Go (TUG), Hospital Anxiety and Depression Scale (HADS), and Falls Efficacy Scale-International (FES-I) were compared at pre-visitation, before and after the 2-week regimen, and again at 4 and 10 months follow-up. In total, 224 patients with PD were included. There were short-term improvements in all outcomes. PDQ-39 was maintained at the same level as pre-visitation after 10 months follow-up. A 2-week multidisciplinary rehabilitation regimen improved short-term mobility, muscle function, and HRQOL in individuals with Parkinson’s disease. HRQOL was maintained after 10 months demonstrating long-term effects.

## 1. Introduction

Parkinson’s disease (PD) is a progressive neurodegenerative disease caused by a deficiency in the neurotransmitter dopamine in basal ganglia [1]. PD affects 128–187 per 100,000 persons with an annual incidence of 20 per 100,000 persons worldwide [2]. Motor and non-motor symptoms are common consequences of the disease, affecting several aspects of daily functioning in individuals with PD (IPD). It has been shown that motor symptoms such as impaired balance and slowness of movement are associated with a high risk of falls [3] and a sedentary lifestyle [4]. Examples of non-motor symptoms are disturbed sleeping pattern, sensory dysfunctions, and cognitive problems such as attention and working memory deficits [5]. Motor and non-motor symptoms are present in various degrees already from the early stages of the disease, having a negative impact on IPD’s ability to manage activities of daily living (ADL) [6,7], work, social interactions, and on their perceived health-related quality of life (HRQOL) in general [8].

It is well-documented that reduced muscle strength in healthy elderly is linked to functional deficits such as impaired walking speed and transfer [9] and that moderate intensity resistance-training and cardiovascular exercise improves muscle function, functional ability, and HRQOL [10,11]. IPD have lower muscle strength and function compared with healthy peers [12,13]; consequently, this has a negative impact on IPD’s ADL and HRQOL [14]. Physical exercise such as resistance-training has also been associated with improved muscle function and HRQOL in IPD [15,16,17,18], due to possible positive disease-modifying effects as suggested by animal data [19,20].

As PD is a disease affecting multiple aspects of life, international PD rehabilitation guidelines [21] recommend a multidisciplinary team approach as best clinical practice. Systematic reviews on multidisciplinary rehabilitation have concluded that lower quality studies show positive short-term effects on gait speed and step length, but these improvements are no longer significant at 4–6 months post intervention [22,23]. However, a recent randomized controlled trial (RCT) has shown that an 8-week multidisciplinary rehabilitation program containing task-oriented physical exercises and cognitive-behavioral training improved motor impairments, balance, ADL, and HRQOL after 1 year in IPD [24]. Another study by Ferrazzoli et al. [25]. has shown that after only a 4-week multidisciplinary rehabilitation program focused on aerobic, motor-cognitive, and intensive rehabilitative treatment, IPD maintained HRQOL at 3 months post-intervention. Currently, it is unknown whether a shorter multidisciplinary rehabilitation approach with a longer follow-up period has a beneficial effect on HRQOL in IPD, and we therefore intended to take a first step towards addressing this knowledge gap in the present study.

To address this limited knowledge, the objective of the present feasibility study was to investigate the effect of a 2-week inpatient multidisciplinary rehabilitation regimen with long-term follow-up at 10 months that focused on improving HRQOL, mobility, and muscle function in IPD. In addition, the study sought to collect data from a short-period of a multidisciplinary rehabilitation regimen primarily consisting of patient education to improve physical and mental health (to induce a sustained behavioral change) for IPD and documents the programs feasibility for future planning of a larger RCT. Data were collected at pre-visitation, before and after the regimen, and at 4 and 10 months follow-up. We hypothesized that IPD would benefit from a 2-week inpatient multidisciplinary rehabilitation regimen by improving mobility, physical function, and health-related quality of life HRQOL with lasting effects.

## 2. Materials and Methods

### 2.1. Participants and Study Design

This study is designed as a prospective cohort feasibility study to assess if a short-term regimen of 2-weeks could have a positive impact on HRQOL. IPD were recruited from a specialized PD outpatient clinic at the Department of Neurology, Bispebjerg University Hospital, Copenhagen, Denmark. When recruited, the IPD’s neurological status was assessed, including the severity of PD as evaluated on Hoehn and Yahr (H and Y) stages of disease 1–5 (H and Y scale describe how PD symptoms progress; in stage 1 the patient experience unilateral involvement, in stage 5 the patient is wheelchair bound unless aided) [21]. Subsequently, the study participants were referred to a “pre-visitation” for a 2-week inpatient rehabilitation program at one of following two rehabilitation centers nearest to their home: Vejlefjord Rehabilitation Center (VRC), Vejlefjord, and Center for Health and Rehabilitation, Danish Association for Rheumatism, Skaelskoer (CST). The “pre-visitation” was held three months prior to the intervention onset and at this timepoint baseline testing was performed.

IPD stayed at the center for 2 weeks, this in order to minimizes logistical problems with transportation and optimize the intensity of the regimen. Inclusion- and exclusion criteria are presented in Table 1.

### 2.2. Ethical Considerations

All participants provided written informed consent before initiation of any study procedures, and the study was complied with the Helsinki Declaration.

### 2.3. Components of the Multidisciplinary Rehabilitation Program

The multidisciplinary team consisted of a neurologist, neuropsychologist, psychologist, sex therapist, speech therapist, physiotherapists, occupational therapists, and a nurse. All team members had expert knowledge in the treatment of PD.

The 2-week multidisciplinary rehabilitation program was delivered sequentially to groups of 8 participants. The main components of the program were (a) a comprehensive introduction to resistance-training and (b) daily training supervised by physiotherapists with the purpose of introducing and testing alternative activities with physical and psychological elements. IPD’s were introduced to Nordic Walking, aqua training in a heated pool, meditation, and dancing.

Furthermore, health promotion initiatives and lectures with a wide variety of relevant issues for IPD were given. A neurologist held a lecture on PD, updated on the newest evidence with concern of treatment, typical symptoms, and prognosis. A neuropsychologist had focus on insight and understanding of the stress load that accompanies the disease; tools were given to handle stress and reduce the impact on daily living. The session contained theoretical presentation and exercises which involved participants. A psychologist gave insight in the emotional reactions with PD, coping with stress, griefs, and crisis. The psychologist also held a special session for the relatives in order for them to gain more knowledge on PD in a closed environment, where they also were able to exchange experiences. Nutrition was reviewed by a nurse with focus on the composition of a nutritious diet. Occupational therapists gave information on assistive devices. Furthermore, they held sessions on coping with tools to discover their own resources and implementing them in daily living. A sex therapist informed on sexuality and cohabitation with advice and guidance on the possibilities for functioning as well as possible challenges that may arise when one party is affected by PD. Lastly, a speech therapist presented knowledge on voice strength and how it might be affected by PD, respiration and communication exercises (see Table 2). Relatives were invited to attend along with their spouses or partners to increase their knowledge about the disease, enable them to support their spouses in managing their everyday life and to network with others in a similar life context. Sessions were scheduled from morning to evening and lasted between 1 h to 1.5-h; shortest sessions were aqua training with 30 active minutes.

### 2.4. Resistance-Training Program

All IPD were given an in-depth introduction to a simple resistance-training program targeting balance, muscle strength, and function. The duration of a single training session was 30–45 min. The program was suitable for training either in the local outpatient physiotherapy clinic, gym, or participants’ homes. The program was designed according to the American College of Sports Medicine guidelines to obtain the best effect and compliance [9].

The resistance-training program was provided/delivered by an experienced physiotherapist three times per week for a total period of 2 weeks (i.e., a total of 6 training sessions). Each training session began with 10-min warm-up exercises of light to moderate intensity on a stationary bike followed by the resistance-training program, which consisted of 3−5 bilateral exercises such as leg press, leg curl, leg extensions push, and pulldown. The participants completed 2−3 sets of each exercise with 1–2-min rest between each set. The repetitions/loads were: Week 1: 12 repetitions with loads corresponding to 15 repetition of maximum (RM). Week 2: 12RM-9RM [10,26]. Patients were instructed to spend 3–4 s completing the concentric phase and 3–4 s completing the eccentric phases, i.e., 6–8 s repetition duration to achieve neural effect and avoid high peak forces [27]. After the 2-week inpatient multidisciplinary rehabilitation regimen, the participants were encouraged to continue their resistance-training exercises-three weekly sessions: 3–4 sets of 10RM-6RM for another 14 weeks (16 weeks in total). The repetitions/loads corresponded to 12RM-10RM in the beginning and approaching 6RM by the end of 14 weeks. All resistance-training sessions were completed with stretching exercises focusing on typically tight flexor muscles: hip-flexors, calf’s, and pectoralis muscles.

All participants received a logbook. To improve compliance for continued training, the participants’ local physiotherapist was informed about the rationale including the evidence behind the PD resistance-training program. Participants received two strength training programs: one program based on training on machines at the gym, and one home-based that could be implemented without machines and equipment such as backpack, bags with water containers and TheraBand (elastic training band). Both programs could be used by the local physiotherapist and were not dependent on advanced training equipment.

In order to accommodate any questions concerning training, participants had the opportunity to get into contact with the study physiotherapist by phone on a weekly basis during the four months follow-up. Furthermore, participants were contacted by a physiotherapist by phone at 5 months and 7.5 months after completion of the multidisciplinary rehabilitation program in order to improve adherence to continued training [28].

### 2.5. Data Collection

**Figure 1:**
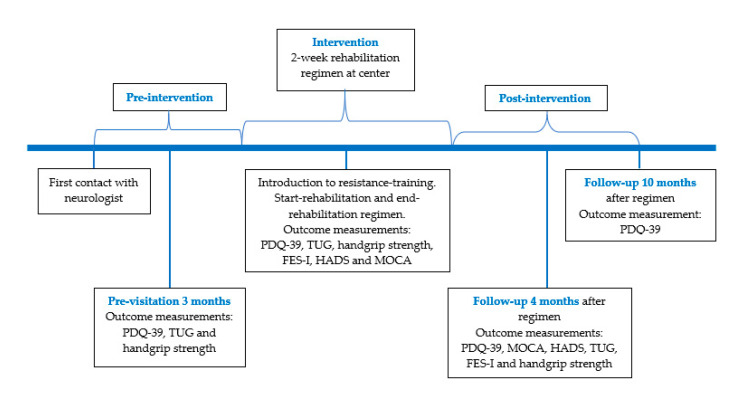
Diagram of the study. First contact: Neurologist. Pre-visitation: Outcome measurements performed at one of the two centers. Intervention period: 2 weeks rehabilitation regimen, a multidisciplinary approach. PDQ-39: Parkinson’s DiseaseQuestionnaire-39, TUG: Timed-up & Go, FES-I: falls-efficacy scale international, HADS: Hospital Anxiety and Depression Scale.

Physical and HRQOL assessments were performed at the following timepoints: pre-visitation, start-rehabilitation regimen, end-rehabilitation regimen, and 4 and 10 months after ended intervention (see Figure 1).

All tests were conducted in the morning and approximately 1 h after medication intake; physical tests were performed at the rehabilitation centers by therapists who were not involved in intervention delivery. Questionnaires were collected by administrative personnel with no other role in the study. All assessors and participants were unaware of earlier assessment results.

### 2.6. Outcome Measures

Primary Outcome: HRQOL using Parkinson’s disease questionnaire 39 (PDQ-39) [29]. PDQ-39 is a widely used and acknowledged disease-specific, self-reported questionnaire. It contains 39 questions covering eight distinct domains: mobility, activities of daily living, emotional wellbeing, stigma, social support, cognition, communication, and bodily discomfort [21]. The score for each question ranges from 0 to 4 points, with higher scores indicating higher levels of perceived problems. A change of ≥1.6 points on the PDQ-39 total score after 6 months represents a minimally clinically important difference [29].

Secondary outcomes:

- Handgrip strength. This assessment was first performed on “the most affected side” and then on “the least affected side”, which contained three trials with a short pause (20–30 s) between each attempt. The mean value of the three trials was calculated and entered in the statistical analysis. A North Coast Digital Hand Dynamometer (Gilroy, CA, USA) was used [30]. Same-day test-retest reliability measurements were conducted in seven patients on both sides and the typical error was below 4%. There was no systematic difference between the two highest measurements (paired t-test). The correlation between the two highest measurements was 0.97.
- Timed-Up-and-Go (TUG) was used to test gait function and balance. Participants were timed as they rise from a chair, walk 3 m, turn, and return to sitting on the chair; assistive devices were allowed if needed [31]. High value in time indicate slow performance or worse functionally mobility. The mean value of the two trials was used for the statistical analysis. Same-day test-retest reliability measurements were conducted on seven IPD’s and typical error was below 7%. There was no systematic difference between first and second measurements (paired *t*-test). The correlation between first and second measurement was 0.95. No clinically important differences in TUG has been determined for IPD; however, the minimal detectable change (95% confidence interval) values range from 3.5 to 11 s [32].
- Hospital Anxiety and Depression Scale (HADS): HADS was administrated by neuropsychologists and used as a screening tool for the identification of anxiety and depression. HADS contains 14 questions: seven questions to assess anxiety and seven to assess depression [21]. High score indicates depression and anxiety. Scoring is from 0 to 3 with a total score ranging from 0 to 21, where low score (0–7) indicates low risk of developing anxiety and depression, possible risk (score 8–10) and high risk (score 11–21).
- Falls Efficacy Scale-International (FES-I): Self-reported questionnaire with 16 questions concerning fear of falling. Item scores range from 1 (no worries) to 4 (very worried) [21]. Total score ranges from 16 to 64 points, where higher scores indicate less fall-related self-efficacy and more concern about falling.

### 2.7. Statistical Analysis

Means, standard deviations (SD), and number of available observations are shown for each of the outcomes at each of the five assessment time points. Raw effect sizes are calculated for the change from pre-visitation, start-rehabilitation regimen to end-rehabilitation regimen, 4 months and 10 months, respectively. A multilevel linear regression model was used to assess adjusted mean differences in outcomes between the different time points. Comparisons were made between the following time points: pre-visitation vs. start-rehabilitation regimen, end-rehabilitation regimen, 4 months and 10 months; start-rehabilitation regimen vs. end-rehabilitation regimen, 4 months and 10 months, end-rehabilitation regimen vs. 4 months and 10 months. The model accounted for the inherent excess correlation between the up to five measurements per patient in the study through a patient random effect. The models were adjusted for potential confounding by gender, age and PD severity (H and Y stage). Data were used from all IPD’s, including those who dropped out. Potential differential dropout was adequately handled with the use of the multilevel model [33]. The significance level was *p* < 0.01. SAS version 9.4 (SAS Institute Inc., Cary, NC, USA) was used for the statistical analysis.

## 3. Results

### 3.1. Participants Characteristics

**Figure 2:**
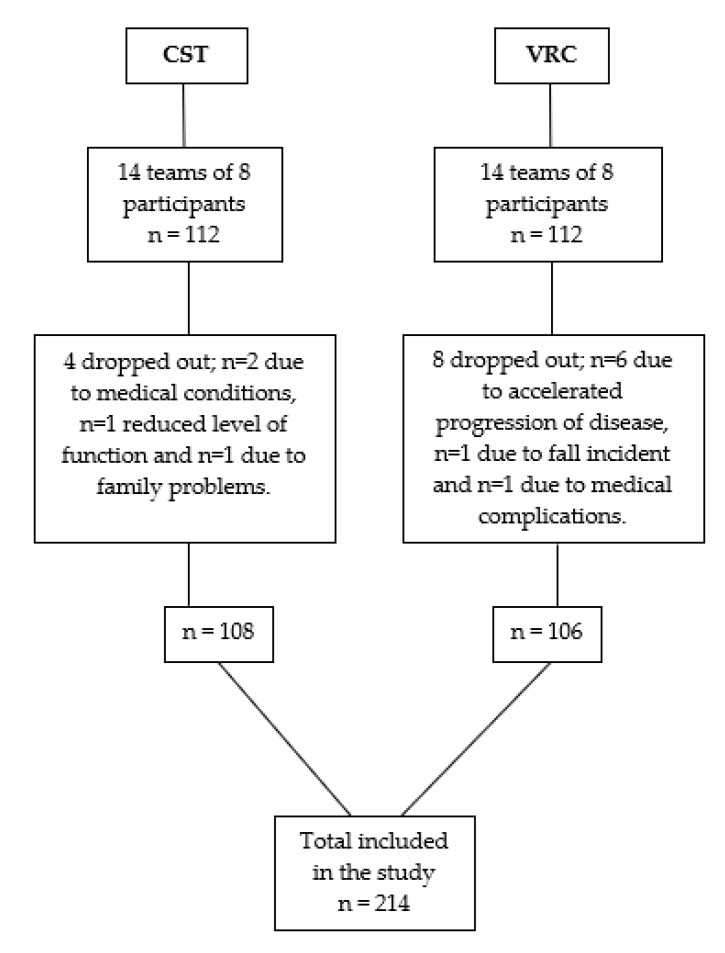
Flowchart of included participants. CST: Center of Health and Rehabilitation, Danish Association for Rheumatism Skaelskoer. VRC: Vejlefjord Rehabilitation Center N: number.

We recruited 224 IDP’s between June 2011 and June 2014, of whom 214 IPD completed the 2-week multidisciplinary rehabilitation program. In total, 112 IPD were treated at VRC and 112 IPD were treated in CTS. A total of 10 drop-outs were encountered. See Figure 2.

Participant characteristics stratified by rehabilitation center are described in Table 3. The intervention the intervention at each center was provided according to the protocol, no deviations was reported.

### 3.2. Outcome Measurements

As illustrated in Table 4 PDQ-39 was reduced by 13% (effect size: 0.3) in score from pre-visitation to end-rehabilitation regimen, at 4 months HRQOL continued to improve (*p* < 0.001). At 10 months PDQ-39 score increased (*p* < 0.001) to the same level as observed at pre-visitation.

Handgrip strength increased by 7% (effect size: 0.2) from 30 to 32 kg (most affected side), (*p* < 0.001) during the 2-weeks regimen. At 4 months, grip strength was improved by 10% (effect size: 0.3) on “the most affected side” from 30 to 33 kg (*p* < 0.001) and 3% (effect size: 0.1) on “the less affected side” from 34 to 35 kg (*p* < 0.001).

TUG reduced by 14% (effect size: 0.4) during the 2-week regimen from 8.5 to 7.3 s (*p* < 0.001). After 4 months, an improvement by 17% (effect size: 0.4) from 8.5 to 7.1 s (*p* < 0.001) was observed.

The HADS score, reduced by 18% (effect size: 0.3) for depression and anxiety during the 2-week program (*p* < 0.001). At 4 months, the depression score was almost back to the level before the multidisciplinary rehabilitation (*p* < 0.01). No other differences were found. Results are presented in Table 4.

For FES-I, no significant differences were found at any time points, but a trend towards a reduced score was observed between pre-visitation and 4 months (*p* = 0.016).

## 4. Discussion

### 4.1. Main Findings

The main findings of this feasibility study were that patients with PD (phase 2–3) improved HRQOL (measured by PDQ-39), mobility, and muscle function (measured by TUG) and handgrip strength on both sides after completion of a 2-week inpatient multidisciplinary rehabilitation program. Furthermore, at 10 months follow-up HRQOL (PDQ-39) was maintained at the same level as at pre-visitation demonstrating long-term effects. These findings suggest that a 2-week inpatient multidisciplinary rehabilitation approach consisting of IPD education with focus on activities that promote HRQOL and physical activity exercise such as using a simple home-based resistance-training program is likely to improve function and sustain long-term HRQOL.

Recently, a systematic review by Johnston and Chu [23] showed that there is limited evidence of short-term benefits in functional outcomes for patients with PD who have attended a multidisciplinary rehabilitation, and after 4–6 months these benefits are no longer significant. Our study demonstrates a significant improvement of the HRQOL (PDQ-39 total-score), (-1.6, p<0.001) during the 2-week multidisciplinary rehabilitation regimen. Furthermore, our patients also demonstrated clinical improvements on the categories of the PDQ-39 such as emotion, stigma, social, and cognition during the 2-week inpatient multidisciplinary rehabilitation and after 4 months of home-based training. Although the PDQ-39 worsened by 3.2 from completion of the 2-week inpatient multidisciplinary rehabilitation regimen compared to 10 months follow-up the PDQ-39 score was still unchanged compared to pre-visitation. The unchanged PDQ-39 score is considered a positive finding indicating that our phase 2–3 IPD’s did not experience any otherwise expected deterioration in HRQOL (PDQ-39) over the 13 months. Our data are somewhat in line with recent findings by Monticone and colleagues demonstrating that multidisciplinary rehabilitative care with emphasis on task-orientated and cognitive behavioral exercises for 8 weeks is useful in changing the course of motor impairments, balance, ADL, and HRQOL, and that these effects last 1-year post intervention [24]. However, in our study we demonstrated that with only 2 weeks of multidisciplinary rehabilitation followed by simple home-based resistance-training program our phase 2–3 patients with IPD had no decline in HRQOL (PDQ-39) after 10 months follow-up compared to pre-visitation. Together, these data indicate that IPD may benefit from the multidisciplinary approach with a focus on progressive strengthening by improving mobility and muscle function on the short-term, but it can also sustain long-term HRQOL improvements.

Handgrip strength has been described as one of most simple methods for assessing general muscle strength and function [34]. In our study, handgrip strength improved on the most affected side after the 2-week multidisciplinary rehabilitation regimen and again after 4 months. On the less affected side a smaller effect on handgrip strength was demonstrated after 4 months.

TUG is a well-proven test and recommended in the Dutch clinical guidelines for PD [21]. In the present study, our patients had clinical improvements of TUG after completion of the 2-week multidisciplinary rehabilitation regimen and again after 4 months. These findings demonstrate that IPD improve and maintain basic mobility (TUG) and function within normal values after both short-term and long term after 2-week multidisciplinary rehabilitation regimen [35].

The HADS score demonstrated a reduction for depression and anxiety after the 2-week multidisciplinary rehabilitation; however, this was not clinically significant as Minimal Clinically Important Difference (MCID) should be beyond 1.3 (depression) and 1.4 (anxiety). Interestingly, the depression component increased level at 4 months was almost at same level as pre-visitation, however this did not apply to the anxiety component. HADS is designed for screening mood disorders in general (non-psychiatric) medical outpatients and aims at distinguishing depression from anxiety [36]. One can therefore argue, if the HADS score is sensitive enough to measure changes over time and that this could be the reason for that no differences was observed.

A previous study by Goodwin [37], has demonstrated that 10-week training intervention consisting of various balance and strength exercises have positive effects on Falls Efficacy Scale-International (FES-I) at 10 weeks and 12 months follow-up, respectively, but not on falls incidents. In our study, no changes were demonstrated at any time points for FES-I. However, from pre-visitation to 4 months a trend towards a reduced FES-I score (*p* = 0.016) was demonstrated, indicating that patients had less fear of falling. Together, this indicates that progressive strength training could influence patients positively in the fear of falling.

### 4.2. Study Limitations

Our study has several limitations. One of them is the lack of a control group or control intervention and it is, therefore, unknown whether a placebo effect was responsible for the greater results. The present prospective cohort feasibility study design was chosen to assess the feasibility of the intervention, relevance of selected outcome measurements and possible effects before performing a larger randomized controlled trial. Therefore, pre-visitation was held 3 months before the 2-week inpatient multidisciplinary rehabilitation period and with long-term follow-up at 4 and 10 months. Furthermore, only PDQ-39 was assessed at 10 months follow-up, but it could also have been interesting to assess outcomes such as functional parameters (e.g., TUG, handgrip strength and gait) and socioeconomic aspects including comorbidities. However, this was not feasible in the present study. Future studies could include these components, but also dosage and intensity of physical training need to be further investigated in order to tailor a sufficient treatment program for the individual patient. Finally, future studies should address the economic benefits with only the 2-week inpatient multidisciplinary rehabilitation regimen and assess the potential cost savings in terms of prevented hospitalization due to falls and injury.

## 5. Conclusions

A 2-week inpatient multidisciplinary rehabilitation program consisting of simple progressive physical exercises, cognitive behavioral therapy, patient education (including Parkinson management and lifestyle changes), and a socially supportive environment improved health-related quality of life (PDQ-39), mobility and muscle function. Health-related quality of life was maintained after 10 months demonstrating long-term effects.

## Figures and Tables

**Table 1:** In- and exclusion criteria.

Inclusion	Exclusion
Diagnosed with PD according to UK Brain Bank Criteria	Psychiatric or geriatric patients
Disease phase 2–3	Patients with day care
Age over 18 years	Medicine or drug addiction
Independent in everyday life	Patients who had attended the rehabilitation offer earlier
Hoehn and Yahr stage 1–3	Other neurological diseases

Disease phase 2: maintenance phase; symptoms are bilateral, minor disability managed effectively by a drug regimen. Disease phase 3: complex phase; more expressed symptoms that become difficult to manage and more complications arise, medications become less effective, additional non-pharmacological approaches are needed.

**Table 2:** Presentation of topics during the 2-week multidisciplinary rehabilitation regimen.

Multidisciplinary Staff	Topic	Purpose
Neurologist	Parkinson’s disease	Insight in PD, symptoms and prognosis.
Neuropsychologist	Stress management	Increase knowledge on stress management; give concrete tools to deal with stress and prevention.
Nurse	Nutrition	Introduction to nutritious diets.
Occupational therapist	Coping	Give insight in ways to change habits and behavior and to find own resources.
Occupational therapist	Assistive devices	Give insight in difference assistive devices.
Physiotherapist	Dancing	Introduction to different types of dancing; inspiration to movement and moving of joy.
Physiotherapist	Mindfulness	To reduce the degree of stress and tension, introduction to meditation and exercises.
Physiotherapist	Nordic walking	Introduction to a physical activity which is feasible in everyday life.
Physiotherapist	Aqua training	Introduction to exercises in water; focus on coordination, mobility and truncus.
Physiotherapist	Resistance training	Introduction to exercises that could be performed at the gym and at home.
Physiotherapist	Theory on training	Increase knowledge on different training activities, effect, intensity and importance of training.
Psychologist	Emotional reactions with PD	Increase the understanding of emotions and PD, special emphasis on stress, crisis and sorrow.
Psychologist	Theme day for relatives	To increase knowledge on PD, talk to other relatives and exchange experiences.
Sex therapist	Sexuality and cohabitation	Advice and guidance on sexuality when a partner is sick with PD, relatives could participate.
Speech therapist	Voice	Increase knowledge of voice, respiration, communication, posture and mimic.

**Table 3:** Participant characteristics.

Variables	Total	CST	VRC
Number of participants (*n*)	214	108	106
Age (Mean ± SD)	66.2 ± 2.8	66.2 ± 8.8	66.1 ± 5.7
Sex (m/f)	96/118	47/59	49/59
Years of disease (Mean ± SD)	7.5 ± 4.2	7.5 ± 3.5	7.5 ± 7.1
Hoehn and Yahr (Mean ± SD)	2.1 ± 1.1	2.1 ± 0.7	2.2 ± 0.7

Number of participants; age, sex, years of disease and Hoehn and Yahr. SD: Standard deviation. Rehabilitation centers: CST: Center for Health and Rehabilitation, Danish Association for Rheumatism, Skaelskoer. VRC: Vejlefjord Rehabilitation Center, Vejlefjord.

**Table 4:** Mean values for outcome measurements.

	PRE		START		END				4 Months				10 Months			
	Mean ± SD	Number (*n*)	Mean ± SD	Number (*n*)	Mean ± SD	Number (*n*)	Effect Size	Sign.	Mean ± SD	Number (*n*)	Effect Size	Sign.	Mean ± SD	Number (*n*)	Effect Size	Sign.
*Primary outcome*																
PDQ-39 (0–100)	26.0 ± 12.0	139	24.3 ± 11.5	196	22.7 ± 11.2	196	0.3	**	22.2 ± 12.0	197	0.3	**	25.9 ± 14.0	178	0.0	§§
*Secondary outcome*																
Grip strength (Kg)																
- Most affected side	30 ± 10.7	142	30 ± 10.8	183	32 ± 10.4	183	0.2	*##	33 ± 12.3	146	0.3	**##	NA			
- Less-affected side	34 ± 11.1	142	34 ± 11.3	183	34 ± 10.5	183	0.0	NS	35 ± 12.4	146	0.1	##	NA			
TUG (Seconds)	8.4 ± 3.1	144	8.5 ± 2.8	205	7.3 ± 3.1	205	0.4	**##	7.1 ± 2.3	157	0.4	**##	NA			
HADS_Depression	5.1 ± 3.4	138	5.0 ± 2.9	191	4.1 ± 3.1	191	0.3	**##	4.7 ± 3.7	153	0.2	§	NA			
HADS_Anxiety	6.7 ± 4.3	138	6.6 ± 4.3	191	5.4 ± 3.9	191	0.3	**##	5.9 ± 3.8	153	0.2	NS	NA			
FES-I	25.3 ± 8.3	123	25.7 ± 8,2	185	25.4 ± 8.1	185	0.0	NS	25.1± 7.5	172	0.0	NS	NA			

Mean values for outcome measurements are presented. PRE: pre-visitation, START: start-rehabilitation regimen, END: end-rehabilitation regimen. Raw effect sizes are calculated for the change from pre-visitation, start-rehabilitation regimen to end-rehabilitation regimen, 4 months and 10 months, respectively. No significant difference was found from pre-visitation and start-rehabilitation, only for PDQ-39. Sign. = Significant difference. Significant from pre-visitation: * (*p* < 0.01), ** (*p* < 0.001), Significant from start-rehabilitation: ## (*p* < 0.001). Significant from end-rehabilitation: § (*p* < 0.01), §§ (*p* < 0.001). “Start-rehabilitation” refers to start of multidisciplinary rehabilitation, whereas “end-rehabilitation” refers to end of rehabilitation that is after 2 weeks. “4 months” and “10 months” refers to 4 months and 10 months, respectively after end-rehabilitation. NS: non-significant. NA: not available. PDQ-39: Parkinson’s Disease Questionnaire-39. TUG: Timed-Up and Go. HADS: Depression: Hospital Anxiety and Depression Scale. HADS_Depression refers to “depression” part of test. HADS_Anxiety refers to “anxiety” of test. FES-I: Falls Efficacy Scale-International.

## References

[1] Huang Z., de la Fuente-Fernandez R., Stoessl A.J. (2003). Etiology of Parkinson’s disease. Can. J. Neurol. Sci..

[2] Khandhar S.M., Marks W.J. (2007). Epidemiology of Parkinson’s disease. Dis. Mon..

[3] Kimmeskamp S., Hennig E.M. (2001). Heel to toe motion characteristics in Parkinsons patients during free walking. Clin. Biomech..

[4] Nimwegen M.V., Speelman A.D., Hoffman-van Rossum E.J., Overeem S., Deeq D.J., Borm G.F., van der Horst M.F., Bloem B.R., Munneke M. (2011). Physical inactivity in Parkinson’s disease. J. Neurol..

[5] Goldman J.G., Litvan I. (2011). Mild Cognitive Impairment in Parkinson’s Disease. Minerva Med..

[6] Gomez-Esteban J.C., Zarranz J.J., Lezcano E., Tijero B., Velasco F., Rouco I., Garamendi I. (2007). Influence of motor symptoms upon quality of life of patients with Parkinson’s Disease. Eur. Neurol..

[7] Aarsland D., Bronnick K., Larsen J.P., Tysnes O.B., Alves G. (2009). Cognitive impairment in incident, untreated Parkinson disease: The Norwegian ParkWest study. Neurology.

[8] Perepezko K., Hinkle J.T., Shepard M.D., Fischer N., Broen M.P., Leentjens A.F., Gallo J.J., Pontone G.M. (2019). Social role functioning in Parkinson’s disease: A mixed-methods systematic review. Int. J. Geriatr. Psychiatry.

[9] Chodzko-Zajko W.J., Proctor D.N., Singh M.A.F., Minson C.T., Nigg C.R., Salem G.J., Skinner J.S. (2009). American College of Sports Medicine position stand. Exercise and physical activity for older adults. Med. Sci. Sports Exerc..

[10] Capodaglio P., Capodaglio E.M., Facioli M., Saibene F. (2007). Long-term strength training for community-dwelling people over 75: Impact on muscle function, functional ability and life style. Eur. J. Appl. Physiol..

[11] Liu C.J., Latham N.K. (2009). Progressive resistance strength training for improving physical unction in older adults. Cochrane Database Syst. Rev..

[12] Cano-de-la-Cuerda R., Perez-de-Heredia M., Miangolarra-Page J.C., Munoz-Hellin E., Fernandez-de-Las-Penas C. (2010). Is there muscular weakness in Parkinson’s disease?. Am. J. Phys. Med. Rehabil..

[13] Schilling B.K., Karlage R.E., LeDoux M.S., Pfeiffer R.F., Weiss L.W., Falvo M.J. (2009). Impaired leg extensor stregnth in indivituals with Parkinson disease and relatedness to functional mobility. Parkinsonism Relat. Disord..

[14] Allen N.E., Sherrington C., Canning C.G., Fung V.S. (2010). Reduced muscle power is associated with slower walking velocity and falls in people with Parkinson’s disease. Parkinsonism Relat. Disord..

[15] Bloomer R.J., Schilling B.K., Karlage R.E., LeDoux M.S., Pfeiffer P.F., Callegari J. (2008). Effect of resistance-training on blood oxidative stress in Parkinson disease. Med. Sci. Sports Exerc..

[16] Dibble L.E., Hale T.F., Marcus R.L., Droge J., Gerber J.P., LaStayo P.C. (2006). High-intensity resistance-training amplifies muscle hypertrophy and functional gains in persons with Parkinson’s disease. Mov. Disord..

[17] Dibble L.E., Hale T.F., Marcus R.L., Gerber J.P., LaStayo P.C. (2009). High intensity eccentric resistance-training decreases bradykinesia and improves Quality Of Life in persons with Parkinson’s disease: A preliminary study. Parkinsonism Relat. Disord..

[18] Uhrband A., Stenager E., Pedersen M.S., Dalgas U. (2015). Parkinson’s disease and intensive exercise therapy—A systematic review and meta-analysis of randomized controlled trials. J. Neurol. Sci..

[19] Speelman A.D., van de Warrenburg B.P., van Nimwegen M., Petzinger G.M., Munneke M., Bloem B.R. (2011). How might physical activity benefit patients with Parkinson disease?. Nat. Rev. Neurol..

[20] Ahlskog J.E. (2011). Does vigorous exercise have a neuroprotective effect in Parkinson disease?. Neurology.

[21] KNGF (2004). KNGF Guidelines for Physical Therapy in Patients with Parkinson’s Disease.

[22] Gage H., Storey L. (2004). Rehabilitation for Parkinson’s disease: A systematic review of avaiable evidence. Clin. Rehabil..

[23] Johnston M., Chu E. (2010). Does attendance at a multidisciplinary outpatient rehabilitation program for people with Parkinson’s disease produce quantitative short term or long term improvements? A systematic review. NeuroRehabilitation.

[24] Monticone M., Ambrosini E., Laurini A., Psy B.R., Foti C. (2015). In-Patient multidisciplinary rehabilitation for Parkinson’s disease: A randomized controlled trial. Mov. Disord..

[25] Ferrazzoli D., Ortelli O., Zivi I., Cian V., Urso E., Ghilardi M.F., Maestri R., Frazzitta G. (2018). Efficacy of intensive multidisciplinary rehahabilition in Parkinson’s disease: A randomised controlled study. J. Neurol. Neurosurg. Psychiatry.

[26] Rhea M.R., Alvar B.A., Burkett L.N., Ball S.D. (2006). A meta-analysis to determine the dose response for strength development. Med. Sci. Sports Exerc..

[27] Aagaard P., Simonsen E.B., Andersen J.L., Magnusson P., Dyhre-Poulsen P. (2002). Increased rate of force development and neural drive of human skeletal muscle following resistance-training. J. Appl. Phsyiol..

[28] King A.C., Haskell W.L., Taylor C.B., Kraemer H.C., DeBusk R.F. (1991). Group- vs home-based exercise training in healthy older men and women. A community-based clinical trial. JAMA.

[29] Peto V., Jenkinson C., Fitzpatrick R. (1998). PDQ-39: A review of the development, validation and application of a Parkinson’s disease quality of life questionnaire and its associated measures. J. Neurol..

[30] Bohannon R.W. (2006). Grip strength predicts outcome. Age Ageing.

[31] Morris S., Morris M.E., Iansek R. (2001). Reliability of measurements obtained with the Timed “Up & Go” test in people with Parkinson disease. Phys. Ther..

[32] Raffertya M.R., Schmidt P.N., Luoc S.T., Lid K., Marrase C., Davis T.L., Guttmang M., Cubillosb F., Simunih T. (2017). Regular Exercise, Quality of Life, and Mobility in Parkinson’s Disease: A Longitudinal Analysis of National Parkinson Foundation Quality Improvement Initiative Data. J. Parkinsons Dis..

[33] Verbeke G., Molenberghs G. (2009). Linear Mixed Models for Longitudinal Data.

[34] Aadahl M., Beyer N., Linneberg A., Thuesen B.H., Jorgensen T. (2011). Grip strength and lower limb extension power in 19-72-year-old Danish men and women: The Health2006 study. BMJ Open..

[35] Bohannon R.W. (2006). Reference Values for the Timed Up and Go Test: A Descriptive Meta-Analysis. J. Geriatr. Phys. Ther..

[36] Leentjens A., Dujardin K., Marsh L., Martinez-Martin P., Richard I.H., Starkstein S.E., Weintraub D., Sampaio C., Poewe W., Rascol O. (2008). Anxiety rating scales in parkinson’s disease; critique and recommendations. Mov. Disord..

[37] Goodwin V.A., Richards S.H., Henley W., Ewings P., Taylor A.H., Campbell J.L. (2011). An exercise intervention to prevent falls in people with Parkinson’s disease: A pragmatic randomised controlled trial. J. Neurol. Neurosurg. Psychiatry.

